# Monocyte-to-lymphocyte ratio as a predictor of outcomes in patients with hepatocellular carcinoma

**DOI:** 10.3389/fonc.2026.1738719

**Published:** 2026-05-08

**Authors:** Weiguang Zhou, Longhui Xiong, Zhijian Li, Zhanqiao Zhang, Jianyang Yang, Huan Cao

**Affiliations:** Department of Hepatopancreatobiliary Surgery, the People's Hospital of Baoan Shenzhen, Shenzhen, Guangdong, China

**Keywords:** hepatocellular carcinoma, meta-analysis, monocyte-to-lymphocyte ratio, prognosis, survival outcome

## Abstract

**Background:**

The monocyte-to-lymphocyte ratio(MLR) as an immune indicator has been found to be associated with the prognosis of various tumors. This study aimed to determine prognostic value of MLR in patients with hepatocellular carcinoma (HCC) by the meta-analysis.

**Methods:**

A comprehensive literature search was conducted in PubMed, Web of Science, and Embase up to July 25, 2025. Survival outcome included overall survival (OS), recurrence free survival (RFS), progression-free survival (PFS), and disease-free survival (DFS) were analyzed.

**Results:**

A total of 17 studies comprising 7113 patients were included in the meta-analysis. The pooled results displayed that high MLR was significantly associated with poor OS (HR:1.57,95% CI:1.22-2.02), RFS(HR:2.40,95%CI:1.73-3.33) and PFS(HR:2.04,95%CI:1.57-2.65).However, high MLR was not associated with DFS(HR:1.01,95%CI:0.86-1.18). The prognostic relevance of MLR in HCC was consistently demonstrated across subgroup analyses.

**Conclusion:**

High MLR was associated with poor prognosis in patients with HCC. MLR may be a valuable and non-invasive prognostic indicator for HCC patients in clinical practice.

## Introduction

According to global cancer statistics from 2022, the liver cancer ranks as the sixth most common diagnosed cancer, with 865,269 new cases of liver cancer reported worldwide (1). Among 36 types of cancer, liver cancer also has the third highest mortality rate (1). Hepatocellular carcinoma (HCC) is the predominant form of primary liver cancer, accounting for approximately 80% of all liver cancer cases (2). Current therapeutic strategies for HCC include liver transplantation, hepatic resection, immunotherapy, radiofrequency ablation, and transarterial chemoembolization (TACE) (3). Nevertheless, HCC is frequently diagnosed at an advanced stage, and even after potentially curative treatments, patients face a high risk of recurrence (4–6), Therefore, accurate prediction of recurrence is crucial for guiding clinical decision-making and improving patient outcomes.

Systemic inflammation plays an important role in cancer biology. Since Virchow first introduced the relationship between inflammation and cancers, many subsequent studies have consistently proved that the occurrence and development of tumors are closely related to inflammatory immune response (7, 8). The inflammatory immune response promotes tumor cell proliferation, angiogenesis and metastasis by upregulating cytokines and producing inflammatory mediators (9). In addition, systemic inflammation can lead to malnutrition, resulting in immunosuppression and a poor prognosis for cancer patients (10). A number of immune inflammation markers have been evaluated for their potential as tumor prognostic biomarkers, such as systemic immune inflammation index(SII), C-reactive protein/albumin ratio(CAR), fibrinogen with neutrophil-lymphocyte ratio (F-NLR) and prognostic nutritional index (PNI) (11–14). However, due to limitations, these indicators are not widely used in digestive system cancers.

Recently, the monocyte-to-lymphocyte ratio(MLR) as a systemic immune-inflammatory marker easily derived from routine blood tests, has been identified as a potential prognostic biomarker in patients with digestive system malignancies receiving immunotherapy (15, 16). However, the criteria for assessing the prognostic value of MLR varied across different cancer types, and its applicability as a prognostic marker in HCC remained unclear. Therefore, we conducted a comprehensive meta-analysis to evaluate the clinical significance of MLR in patients with HCC.

## Material and methods

### Search strategy

A comprehensive literature search was conducted in PubMed, Embase, and Web of Science for studies published up to July 25, 2025. The following search terms were used: “monocyte-to-lymphocyte ratio” OR “Monocyte to Lymphocyte Ratio” OR “monocyte-lymphocyte ratio” AND “hepatocellular carcinoma” OR “hepatic carcinoma” OR “hepatoma” OR “liver cancer” OR “HCC” AND prognosis OR prognostic OR survival OR outcome. The search was limited to articles published in English. Titles, abstracts, full texts, and reference lists were thoroughly screened to identify eligible studies.

### Inclusion and exclusion criteria

Three researchers independently conducted the literature search. The inclusion criteria were as follows: (1) investigated the association between MLR and survival outcomes in patients with HCC. (2) provided sufficient data to calculate the hazard ratios (HRs) and 95% confidence intervals (CIs). The exclusion criteria were as follows: (1) lacked sufficient data to calculate HRs and 95% CIs; and (2) abstracts, case reports, reviews and letters.

### Data extraction and quality assessment

Two independent researchers reviewed each included article and extracted data from the included studies. When discrepancies or uncertainties arose during the data extraction process, a third independent researcher was consulted to arbitrate and ensure the accuracy and consistency of the final data. Relevant data was extracted from each study, including the first author’s name, year of publication, country, tumor type, sample size, source of HR, follow-up time, cut off value of MLR, exclusion criteria, confounding factors, treatment methods, analysis type and survival outcomes. Multivariate analyses were prioritized, as they account for potential confounding factors. When HRs and corresponding 95% CIs were not directly reported in the original studies, they were estimated from Kaplan-Meier survival curves using the methods described by Tierney et al (17). The quality of each study was evaluated using the Newcastle–Ottawa Quality Assessment Scale (NOS) (18). NOS scores of 0–3, 4–6, and 7–9 were considered indicative of low, moderate, and high methodological quality, respectively.

### Statistical analysis

All statistical analyses were conducted using STATA version 12.0 (StataCorp, College Station, TX, USA). HRs and their corresponding 95% CIs were used to estimate the pooled data. A fixed-effects model was applied when heterogeneity was low (I²< 50%), while a random-effects model was used when heterogeneity was substantial (I²≥50%). Subgroup analyses were performed to further explore the prognostic value of MLR in HCC. Sensitivity analysis was used to assess the robustness of the results. Publication bias was evaluated using Begg’s test, Egger’s test and trim-and-fill method (19). A p-value < 0.05 denoted statistical significance.

## Results

### Search results

**Figure 1:**
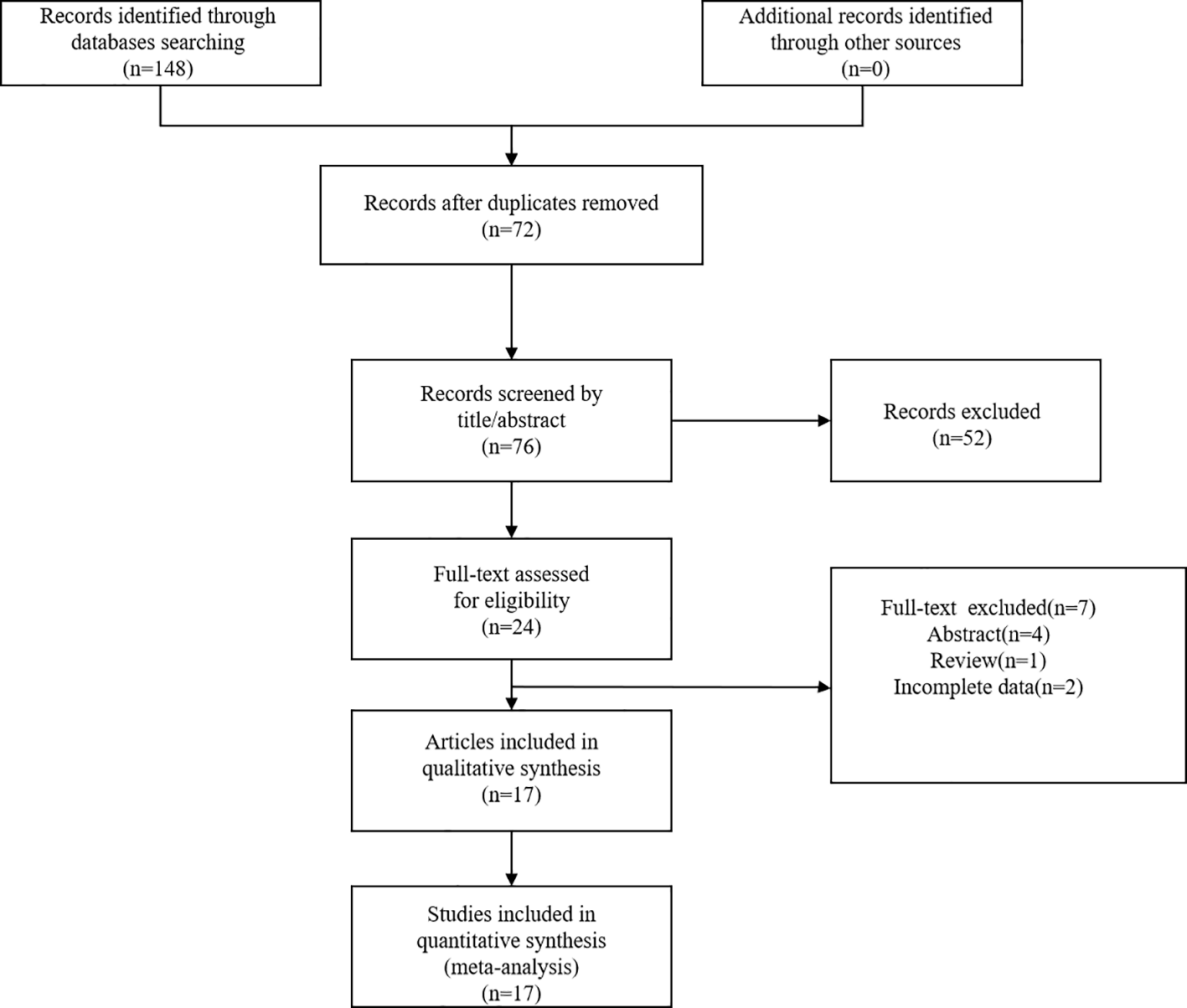
Flow diagram for study screening and selection processes.

A total of 148 articles were initially identified through the systematic literature search. After removing 72 duplicate records, 76 articles remained for further screening. Based on title and abstract review, 52 articles were excluded. Of the remaining 24 articles that investigated the prognostic value of MLR in HCC, 7 articles did not meet the inclusion criteria. Finally, 17 articles published between 2016 and 2025 were included in the final analysis (20–36). The flow diagram of the literature search was shown in **Figure 1**.

### Study characteristics

A total of 7113 patients with sample sizes ranging from 124 to 1502 participants were included in the meta-analysis. 16 studies were conducted in China, 1 study were conducted in Brazil. 13 studies reported overall survival (OS), 3 studies displayed disease-free survival (DFS), 6 studies covered recurrence free survival (RFS), and 3 studies analyzed progression-free survival(PFS). The NOS scores of the included studies ranged from 6 to 8, with a mean score of 7.41, indicating moderate to high methodological quality. Detailed study characteristics were presented in **Table 1**.

**Table 1:** Basic information of included studies.

Study	Year	Country	Study type	Sample	Treatment methods	Analysis type	Survival analysis	Follow-up time	Cut-off value	Exclusion criteria	Confounders	Source of HR	NOS score
Cui (20)	2023	China	R	218	Surgery	MVA	OS,DFS	Median 39.4	0.45	Combined acute infection; preoperative diagnosis of hypersplenism; incomplete data	BMI, tumor number, differentiation, hepatic encephalopathy	Reported	8
Fu (21)	2018	China	R	150	Surgery	MVA	OS	Median 41	0.27	NR	Gender, age, HBsAg, Child-Pugh Class, tumor number, differentiation	Reported	7
Guo (22)	2020	China	R	407	Interventional treatment	MVA	OS	NR	0.27	Active infection; severe coagulation disorders; serious hemorrhage; receiving any medication that might seriously infected inflammatory markers or loss of regular follow-up.	Gender, age, TNM stage, BCLC staging, portal vein involvement	Reported	7
Liao (23)	2016	China	R	387	Surgery	MVA	OS,RFS	Median 44	0.3	NR	Gender, age, liver cirrhosis, HBsAg, tumor number, tumor differentiation, tumor size, TNM stage	Reported	8
Liu (24)	2023	China	R	104	Immunotherapy	UVA	OS,PFS	NR	0.28	Other concurrent malignancy; recent infection; untreated HIV infection or (auto)immune diseases; anti biotic use; corticosteroid administration	NR	Reported	7
Mai (25)	2022	China	R	1502	Surgery	MVA	OS,DFS	NR	0.3	Received other therapies before hepatectomy	Gender, age, Child–Pugh grade, HBsAg, tumor number, tumor size, BCLC stage	Reported	8
Mai (26)	2024	China	R	1039	Surgery	MVA	OS,DFS	NR	0.3	Other anti-cancer therapies before hepatic resection	Gender, age, HBsAg, tumor number, tumor size, BCLC stage	Reported	8
Mao (27)	2020	China	R	128	Surgery	MVA	OS,RFS	NR	NR	Preoperative infection	Tumor burden, viral load	Reported	8
Silva (31)	2022	Brazil	R	161	Surgery	UVA	OS	Median 62	1.75	Previous treatment; infection; use of preoperative therapeutic antibiotics or corticosteroids	Portal hypertension, vascular invasion, AST, ICU stay	Extracted	7
Wang (32)	2022	China	R	606	TACE combined with ablation	MVA	OS,RFS	Median 59.4	0.44	Infection or other inflammation; Child–Pugh class C; accompanied by other malignancies	Age, sex, hypertension, diabetes mellitus, smoking history, cirrhosis ,Child–Pugh grade, tumor number, tumor size, AST, ALT, bilirubin, albumin, INR	Reported	8
Zhang (34)	2024	China	R	1496	Surgery	MVA	OS	NR	NR	Presence of infection or inflammation; disorders in the coagulation function; other serious metabolic diseases and immune system diseases	Abumin, AST, globulin, prothrombintime, glutamyltrans ferase, INR, bilirubin, Child-Pugh grade	Reported	7
Zhu (36)	2018	China	R	142	Non-surgery	MUA	OS,PFS	NR	0.35	NR	Age, gender, BCLC grade, albumin, ALT, AST, bilirubin	Reported	8
Wang (29)	2024	China	R	100	Surgery	MVA	OS	NR	0.3	Age <18 years ;intrahepatic cholangiocarcinoma or other malignancies	Gender, age, BMI, Child-Pugh grade, tumor number, tumor size, TNM stage	Reported	7
Mao (28)	2021	China	R	166	Surgery	MVA	RFS	Median 20	NR	Incomplete clinical and follow-up data	ALBI grade, INR, tumor number, tumor size, portal vein tumor thrombosis	Reported	6
Wu (33)	2021	China	R	161	Surgery	MVA	RFS	NR	0.25	Infection or systemic inflammatory disease; other malignant tumors; immune or hematological disease ;Preoperative treatment with immunosuppressants	Age, sex, AST, ALT, bilirubin, albumin, INR, differentiation	Reported	7
Zhao (35)	2023	China	R	124	HAIC	MVA	PFS	NR	NR	Incomplete follow-up data; Other malignant tumors	Tumor number, tumor size, ALBI grade, BCLC stage	Reported	6
Zhou (30)	2025	China	R	222	Surgery	MVA	RFS	Median 70.3	0.23	History of palliative hepatectomy; missing or incomplete data; history of preoperative anti-HCC treatment	Gender, age, albumin, AST, ALT, Child-Pugh grade	Reported	6

R, Retrospective; OS, overall survival; DFS, disease-free survival; RFS, recurrence free survival; PFS, progression-free survival; NOS score, Newcastle-Ottawa Scale score;MVA: multivariable analysis; UVA: univariable analysis;TACE, transcatheter arterial chemoembolization;HAIC,hepatic arterial infusion chemotherapy; NR,not reported;.

### Association between high MLR and OS

**Figure 2:**
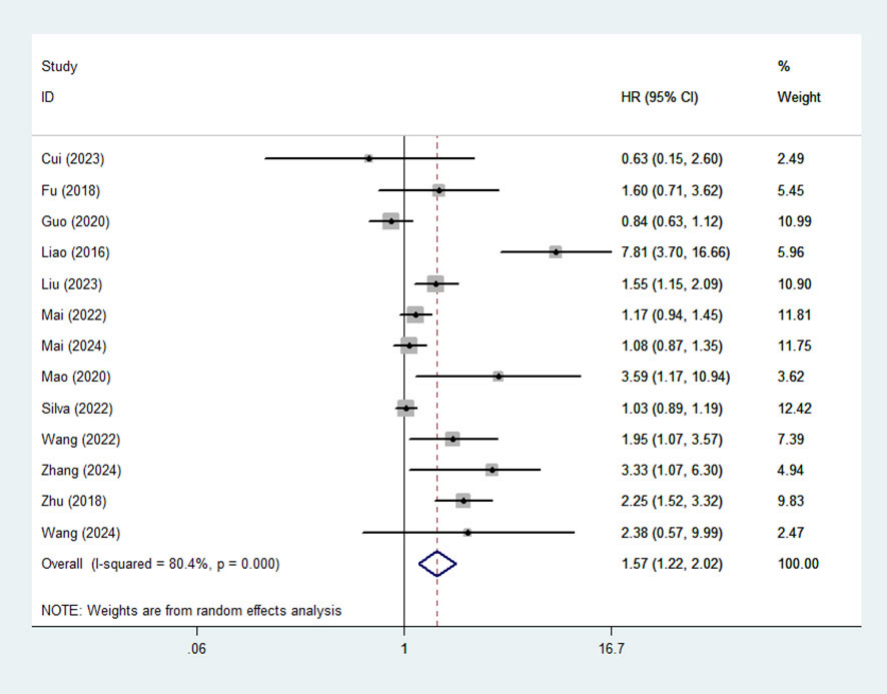
Forest plot of the association between high MLR and OS.

13 studies assessed the association between MLR and OS in patients with HCC. A random-effects model was applied to calculate the pooled HRs due to substantial heterogeneity (I² = 80.4%). The meta-analysis revealed that high MLR was significantly associated with poor OS (HR:1.57,95% CI: 1.22-2.02) (**Figure 2**).

### Subgroup analysis and meta-regression for OS

We further conducted subgroup analyses based on country, treatment method, analysis type and sample size. The results were shown in **Table 2**. We found that high MLR was an unfavorable prognostic marker in the subgroup of China (HR: 1.71; 95% CI: 1.27-2.30), surgery(HR:1.63; 95% CI: 1.18-2.24), sample size(>500) (HR:1.36; 95% CI:1.01-1.85),sample size(<500) (HR:1.71; 95% CI:1.16-2.51) and MVA (HR:1.75; 95% CI:1.25-2.46). Meta-regression indicated that treatment method might be the most significant source of heterogeneity.

**Table 2:** Subgroup analysis and meta-regression for OS.

Factors	Studies	HR(95%)	P	Heterogeneity			Meta-regression		
				I^2^	P	Model	Tau2	Adj R2 (%)	P
Country							0.304	-5.07	0.383
China	12	1.71(1.27-2.30)	<0.001	75	<0.001	Random			
Brazil	1	1.03(0.89-1.188)							
Treatment method							0.03	67.5	0.041
Non-surgery	4	1.51(0.94-2.41)	0.086	84.3	<0.001	Random			
Surgery	9	1.63(1.18-2.24)	0.003	79.7	<0.001	Random			
Sample size							0.32	-13.66	0.791
>500	4	1.36(1.01-1.85)	0.047	84.8	0.036	Random			
<500	9	1.71(1.16-2.51)	0.007	84.8	<0.001	Random			
Analysis type									
MVA	11	1.75(1.25-2.46)	0.0001	81.1	<0.001	Random	0.319	-9.03	0.45
UVA	2	1.24(0.83-1.84)	0.296	82.7	0.016	Random			

MVA, multivariable analysis; UVA, univariable analysis.

### Association between high MLR and DFS/PFS/RFS

**Figure 3:**
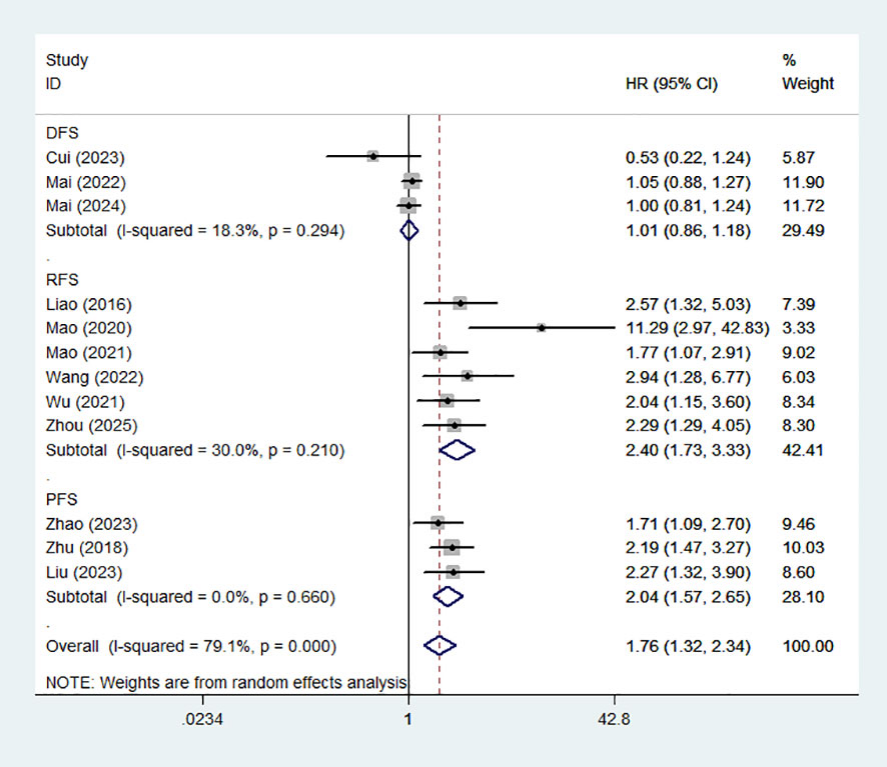
Forest plot of the association between high MLR and DFS/PFS/RFS.

12 studies documented the association between MLR and DFS/PFS/RFS in patients with HCC. The meta-analysis indicated that high MLR was associated with unfavorable RFS(HR:2.40,95%CI:1.73-3.33) and PFS(HR:2.04,95%CI:1.57-2.65).However, high MLR was not associated with DFS(HR:1.01,95%CI:0.86-1.18).The corresponding forest plot was presented in **Figure 3**.

### Sensitivity analysis

**Figure 4:**
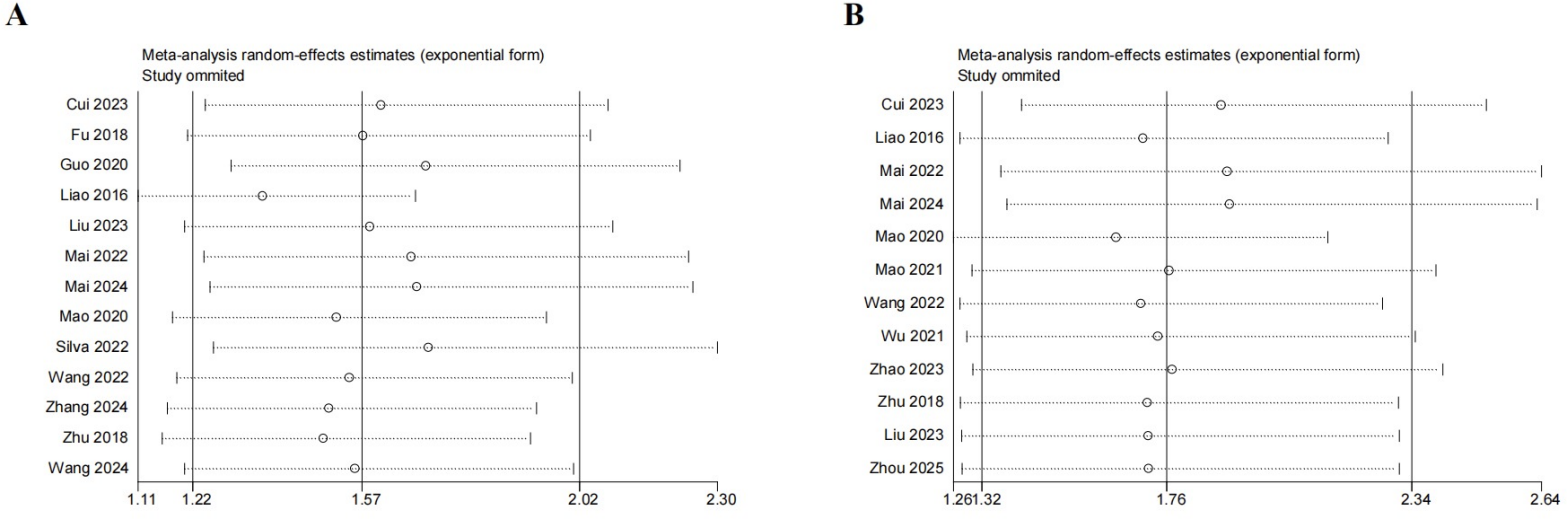
Funnel plot of sensitivity analysis. **(A)** Sensitivity analysis for OS. **(B)** Sensitivity analysis for DFS/PFS/RFS.

Sensitivity analysis was implemented by sequentially excluding individual studies to assess the robustness of the pooled results. The results remained consistent, indicating that meta-analysis of OS(**Figure 4A**) and DFS/PFS/RFS(**Figure 4B**) were relatively robust despite the predominance of retrospective studies.

### Publication bias

**Figure 5:**
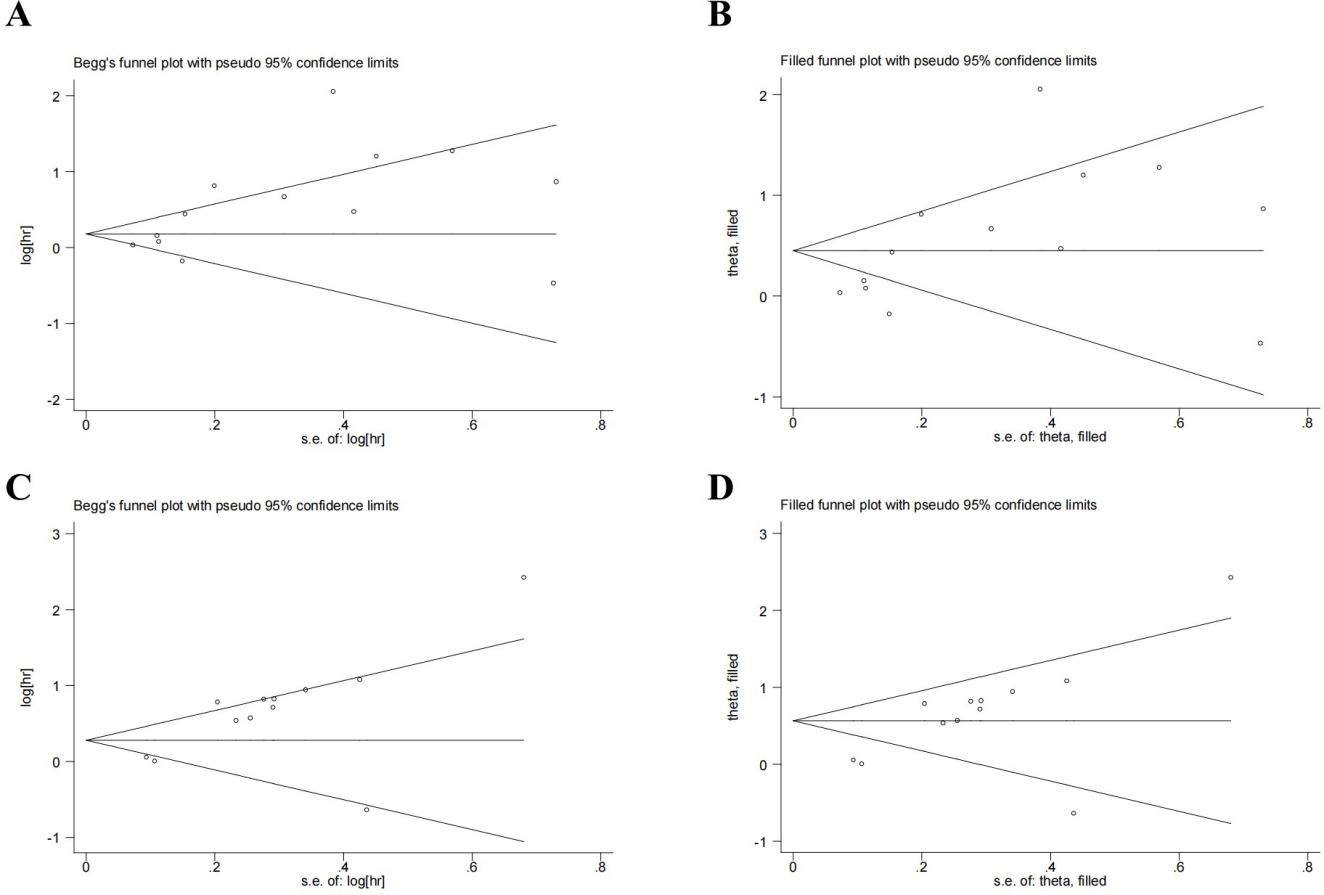
Publication bias. **(A)** Publication bias for OS. **(B)** Trim-and-fill method for OS. **(C)** Publication bias for DFS/PFS/RFS. **(D)** Publication bias for DFS/PFS/RFS.

Begg’s test and Egger’s test were used to evaluate the publication bias. P value of Begg’s test and Egger’s test for OS were 0.200 and 0.019, respectively (**Figure 5A**), indicating a potential publication bias. Although some degree of publication bias was detected, the trim-and-fill method indicated that the overall results remained unaffected (HR: 1.571; 95% CI: 122-2.02) (**Figure 5B**). P values of Begg’s and Egger’s tests for DFS/PFS/RFS were 0.15 and 0.004, respectively (**Figure 5C**). The result for DFS/PFS/RFS was stable through the trim-and-fill method(HR:1.758,95%CI:1.32-2.34).

## Discussion

To the best of our knowledge, this was the first meta-analysis to assess the prognostic significance of MLR in HCC. A total of 17 studies involving 7113 patients were included. Our findings suggested that high MLR was significantly associated with unfavorable OS,RFS and PFS in HCC patients. Furthermore, sensitivity analysis, Begg’s test and Egger’s test confirmed the robustness and reliability of MLR as a prognostic biomarker in patients with HCC. A previous literature reported another indicator of the combination of lymphocyte and monocyte, namely the prognostic value of the lymphocyte-to-monocyte ratio(LMR) in HCC (37). They found that the LMR was associated with DFS and RFS, but not with OS. Our research suggested that MLR was associated with OS,RFS and PFS of HCC patients. Therefore, we believed that MLR may be more suitable than LMR as an effective prognostic marker for HCC.

The prognostic significance of MLR may vary across different treatment modalities. Patients undergoing surgical resection are typically diagnosed at relatively earlier stages and often have better preserved liver function and immune status. In this population, systemic inflammatory markers such as MLR may more accurately reflect the host immune response against tumor progression. In contrast, patients receiving TACE are often characterized by more advanced tumor burden or unresectable disease, accompanied by a more complex inflammatory and immunosuppressive tumor microenvironment. Moreover, TACE itself can induce tumor necrosis and inflammatory responses, which may further influence circulating inflammatory biomarkers. Therefore, the prognostic value of MLR may differ between surgical and nonsurgical treatment group, highlighting the importance of considering treatment modality when interpreting the clinical significance of inflammatory markers in HCC patients.

Another important issue that warranted further consideration was the lack of a standardized cut-off value for the MLR. Across the included studies, the cut-off values varied considerably, which may limit the direct clinical applicability and comparability of results. This variability was likely attributable to differences in patient characteristics, including tumor stage, underlying liver function, and treatment modalities, as well as the use of different statistical approaches (ROC curve analysis or median value) to determine optimal cut-off points. Moreover, MLR was inherently dynamic and can be influenced by various clinical conditions, including infection, liver function and prior treatments. These factors may further contribute to inter-study heterogeneity and complicate the establishment of a universally applicable threshold. Given these limitations, the prognostic value of MLR should be interpreted with caution in clinical practice. Future well-designed prospective and multicenter studies were needed to establish standardized and clinically meaningful cut-off values. In addition, the integration of MLR with other established prognostic indicators or composite scoring systems may improve its predictive accuracy and enhance its utility in individualized risk stratification for patients with HCC.

MLR based on monocyte and lymphocyte count was identified as a valid prognostic indicator for many cancers. Monocytes and lymphocytes, as immune cells, play a significant role in the tumor immune response. MLR combined with monocyte and lymphocyte can better reflect inflammatory response and cancer immune nutritional status. MLR overcomes the adverse effects of monocyte or lymphocyte alone and effectively improves its predictive efficacy in cancer patients.

Monocytes play an important role in shaping the tumor microenvironment and influencing tumor progression. Circulating monocytes can be recruited into tumor tissues through chemokine-mediated signaling pathways (38). After infiltrating the tumor microenvironment, these monocytes can differentiate into tumor-associated macrophages (TAMs), which are considered one of the most abundant immune cell populations within tumors. TAMs are widely recognized for their tumor-promoting functions and contribute to cancer progression through multiple mechanisms (39). TAMs can promote tumor angiogenesis by secreting pro-angiogenic factors such as vascular endothelial growth factor (VEGF), thereby facilitating tumor growth and providing nutrients and oxygen to rapidly proliferating cancer cells (40). TAMs contribute to tumor invasion and metastasis by releasing matrix metalloproteinases, which degrade the extracellular matrix and enable tumor cells to invade surrounding tissues (41). In addition, TAMs exert immunosuppressive effects within the tumor microenvironment by producing cytokines such as interleukin-10 and transforming growth factor-β, which inhibit cytotoxic T-cell activity and promote immune evasion by tumor cells (42). Moreover, TAMs can interact with other immune cells, including regulatory T cells and myeloid-derived suppressor cells, further enhancing the immunosuppressive microenvironment (43). Therefore, elevated peripheral monocyte counts may reflect increased recruitment of monocytes into tumor tissues and enhanced TAM-mediated tumor-promoting activity. Studies showed that elevated peripheral blood monocyte counts have been associated with unfavorable prognoses in cancers such as oral cavity, cervical, colorectal and prostate cancers (44, 45).

Conversely, lymphocytes play a central role in anti-tumor immune responses. Cytotoxic CD8^+^T cells are capable of directly recognizing and eliminating malignant cells, while CD4^+^ helper T cells coordinate adaptive immune responses against tumor antigens (46, 47). A decreased lymphocyte count may indicate impaired immune competence and weakened tumor immune surveillance (48). Previous studies have shown that reduced lymphocyte levels are associated with poor survival outcomes, while higher lymphocytes show favorable survival outcomes for cancer patients (49–51).

Taken together, a high MLR reflects both increased monocyte-mediated tumor-promoting inflammation and decreased lymphocyte-mediated anti-tumor immunity. This imbalance between pro-tumor inflammatory responses and anti-tumor immune defenses may contribute to tumor progression, metastasis, and unfavorable clinical outcomes. Therefore, MLR may serve as a convenient and non-invasive biomarker that reflects the immune status of cancer patients and provides prognostic information in clinical practice.

There were some limitations in the study. Firstly, all articles had small sample sizes. Secondly, all studies were retrospective analysis, which may introduce potential selection bias. Therefore, the results should be interpreted with caution. Thirdly, the cut-off value of MLR varied among studies, which might contribute to heterogeneity. Fourthly, the HRs and CIs values of some studies were extracted from the survival curves. Fifthly, although sensitivity analyses suggested that the results were relatively stable, potential confounding factors such as differences in treatment strategies, patient characteristics, and follow-up duration cannot be completely excluded. Sixthly, most studies included in the meta-analysis were conducted in China. More studies from other regions were warranted. Additionally, MLR may be influenced by various clinical factors, such as infection status, liver function, and prior treatments. However, these potential confounders were not consistently reported across the included studies and therefore could not be systematically summarized or analyzed in this meta-analysis. This limitation may contribute to inter-study heterogeneity and should be considered when interpreting the results. Finally, publication bias existed in the study.

Although there were some defects, the study also had some strengths. Firstly, this was the first meta-analysis to assess the relationship between MLR and prognostic outcome in HCC. Secondly, the combined results were stable through sensitivity analysis. Thirdly, the trim-and-fill method found that the results were unaffected by the publication bias. Finally, as a convenient serum marker, MLR can dynamically monitor the prognosis and therapeutic effect of HCC patients.

In conclusion, our study suggested that high MLR was significantly associated with poor survival outcomes in patients with HCC. Given its accessibility, non-invasive nature, and capacity for dynamic monitoring, MLR holds promise as a prognostic biomarker for HCC. It can help doctors better identify high-risk patients so they can treat them more effectively. However, due to the shortcomings, further prospective and multicenter studies were warranted to validate our findings.

## Data Availability

The original contributions presented in the study are included in the article/Supplementary Material. Further inquiries can be directed to the corresponding author.
